# Tumor glycosylation engages CD301b-mediated myeloid regulation in breast cancer

**DOI:** 10.21203/rs.3.rs-9347231/v1

**Published:** 2026-05-05

**Authors:** Ahmet Ozdilek, Amy V. Paschall, Fathima Z. Nawaz, Afaq M. M. Niyas, Fikri Y. Avci

**Affiliations:** Emory University; Emory University; Emory University; Emory University; Emory University

## Abstract

Aberrant tumor glycosylation can alter immune recognition; however, the specific influence of glycan-lectin interactions on tumor progression remains poorly understood. Here, we identify the C-type lectin receptor CD301b (encoded by *Mgl2*) as a regulator of immune activity within the breast tumor microenvironment (TME) and identify a cross-species myeloid regulatory program associated with its human ortholog CLEC10A. Using a murine triple-negative breast cancer model, we demonstrate that tumors expressing the Tn glycoantigen grow more rapidly, and this growth is facilitated by CD301b^+^ immune cells. Depletion or genetic loss of CD301b markedly suppressed tumor growth, indicating that CD301b promotes tumor progression, potentially through myeloid-tumor interactions. Phenotypic analyses revealed that tumor-infiltrating CD301b^+^ cells are predominantly type 2 conventional dendritic cells (cDC2s) and exhibit IL-10 expression within the TME. Transcriptomic profiling of tumors developed in *Mgl2*-KO mice revealed a shift toward an inflammatory, interferon-dominant transcriptional state, consistent with altered antitumor immune programming. Single-cell RNA sequencing of human breast cancers revealed that CLEC10A is expressed in cDC2 and select macrophage subsets. Additionally, CLEC10A-positive cDC2 and macrophages share a transcriptional state characterized by enhanced antigen presentation and immune-regulatory functions compared to CLEC10A-negative cells. Together, these findings support a relationship between tumor glycosylation and CLEC10A/CD301b-associated myeloid regulation, highlighting this axis as a potential target for reprogramming the breast tumor immune microenvironment.

## Introduction

A major setback observed in many cancers arises from immune modulation within the tumor microenvironment (TME), preventing effective anti-cancer responses. Vaccines, CAR T cells, and checkpoint blockade prime the immune system against cancer-associated antigens, promoting cancer cell destruction through immune cytotoxicity ^1^. Immune therapies for breast cancer remain largely ineffective due to the ability of the tumor to grow unimpeded and eventually metastasize. Immune regulation can occur through cytokine secretion, signaling mechanisms, or checkpoints (such as through PD-1) ^2–5^. During the malignant transformation of a mammalian cell, a dramatic and aberrant modification of cellular glycosylation is observed. Tumor-associated carbohydrate antigens, or TACAs, can induce immune suppression, allowing cancer cells to evade immune cells ^6–8^. Lectins are carbohydrate-binding proteins that function as receptors for immune cells and can activate immune regulatory pathways through their interactions with TACAs ^9–11^. Similarly, modulating immune profiles in the TME by engaging sialic-acid-binding immunoglobulin-like lectins (Siglecs) is a known TACA mechanism ^12–14^. Thus, elucidating the roles of lectins in regulating the immune response within the TME is becoming increasingly important.

Tn antigen is a TACA ranked as a high-priority cancer-associated antigen based on its antigenicity and oncogenicity ^11,15–19^. Tn is a truncated form of the cell surface O-glycan, consisting of the terminal O-linked N-acetylgalactosamine (GalNAc) attached to serine or threonine. The aberrant glycosylation associated with Tn may occur when the enzyme responsible for O-glycan elongation, T-synthase or its associated chaperone, Cosmc (C1GALT1C1), becomes functionally inhibited ^20,21^. Tn-expressing mucin 1 (MUC1) has been associated with breast cancer cells ^22,23^, especially in triple-negative breast cancer ^24,25^. Modulating MUC1 expression or Tn glycosylation can inhibit tumor growth ^26–28^. However, developing immune responses against Tn-MUC1 has been problematic ^29^.

Human CLEC10A, also known as macrophage galactose-type lectin (MGL), is a C-type lectin receptor (CLR) that binds to Tn antigen on the surface proteins in humans ^19,30^. In mice, the Tn-recognizing homolog of the human CLEC10A is CD301b, also known as MGL2 ^19,31^. CD301b is primarily expressed by myeloid cells such as dendritic cells (DCs) and macrophages ^17^. Previously, CD301b-expressing myeloid cell populations have been linked with immunosuppressive responses ^16,17,19,32–34^. DCs and macrophages can suppress the proliferation of CD4^+^ effector T lymphocytes through the interaction of MGL with terminal GalNAc residues on CD45 expressed by T cells ^17^. This cell-specific glycosylation of CD45 provided an immunoregulatory pathway, mediated by MGL, thereby controlling effector T cell function. In another study, CD301b^+^ dendritic cells suppressed T follicular helper cells and antibody responses to protein antigens ^32^. Recently, an immunosuppressive DC subset expressing CD301b was shown to accumulate at secondary sites and promote metastasis in pancreatic cancer ^34^ and lung cancer ^35^.

Breast cancer-focused studies further support a functional Tn-CD301b/CLEC10A axis by showing that ligand display can be induced by clinically relevant tumor-cell perturbations, including endocrine manipulation (e.g., tamoxifen exposure), oxidative stress, and DNA damage, linking stress signaling to increased presentation of truncated O-glycan ligands^36^. In the same context, receptor-ligand readouts have been associated with breast cancer outcomes, suggesting that these glycan-lectin interactions reflect biologically meaningful variation in tumor-immune dynamics^36^. Complementing these observations, transcriptomic and immunoinformatics analyses across breast cancer cohorts and pan-cancer datasets indicate that CLEC10A expression tracks immune infiltration patterns and immune regulatory networks and is frequently associated with a more favorable prognosis, supporting further investigation into how tumor glycan presentation intersects with CLEC10A/CD301b signaling in vivo^37–39^.

In this study, we investigated the role of CD301b in a murine model of triple-negative breast cancer and found that the loss of CD301b expression significantly restricted tumor growth. Within the tumor microenvironment, CD301b-expressing immune cells were identified as type 2 conventional dendritic cells. To further explore the mechanisms underlying the observed tumor growth restriction, we performed bulk RNA sequencing (bulk RNA-seq) on murine tumors, which showed heightened inflammatory immune responses when CD301b was absent. Analysis of publicly available single-cell RNA sequencing (scRNA-seq) datasets revealed CLEC10A-expressing myeloid cell populations in human breast cancer tissues, suggesting that this regulatory axis extends beyond the murine model. Here, we examine whether the Tn-CD301b/CLEC10A axis links tumor glycosylation to a conserved myeloid regulatory program in breast cancer.

## Results

### CD301b/Tn Axis Impacts Tumor Growth.

We first aimed to investigate the relationship between CD301b^+^ immune cells and Tn glycoantigen-expressing breast cancer cells. We used a CRISPR-Cas9 gene editing model to knock out Cosmc expression in AT3 murine breast cancer cells. Cosmc is a chaperone essential in elongating the core O-glycan beyond the truncated Tn form of α-GalNAc ^20,21,40^. When the Cosmc function/expression is disrupted, elongation of the O-glycan is not observed; instead, the Tn antigen is observed at significantly higher levels. After disrupting Cosmc expression in these cell lines, we confirmed increased Tn cell surface expression through flow cytometry using a reBaGs6 IgM antibody (Suppl. Figure 1A) ^41^, a biotinylated VVL lectin coupled with fluorescent streptavidin (Suppl. Figure 1B), and a recombinant biotinylated Mgl2 carbohydrate binding domain (CBD)-GFP fusion protein coupled with streptavidin (Suppl. Figure 1C). All staining methods indicated significantly higher Tn expression on the *Cosmc* KO cell line. We also isolated RNA from each cell line and confirmed decreased Cosmc expression in the Tn^hi^ cell line through qPCR using *Cosmc* primers (Suppl. Figure 1D). We then tested whether knocking out Cosmc expression changes the proliferation rate of the AT3 breast cancer cells *in vitro*. After culturing both cell lines at the same concentrations for three days, we observed no significant differences in cell proliferation between the two lines (Suppl. Figure 1E), indicating that Tn expression alone does not promote cancer cell growth.

**Figure 1:**
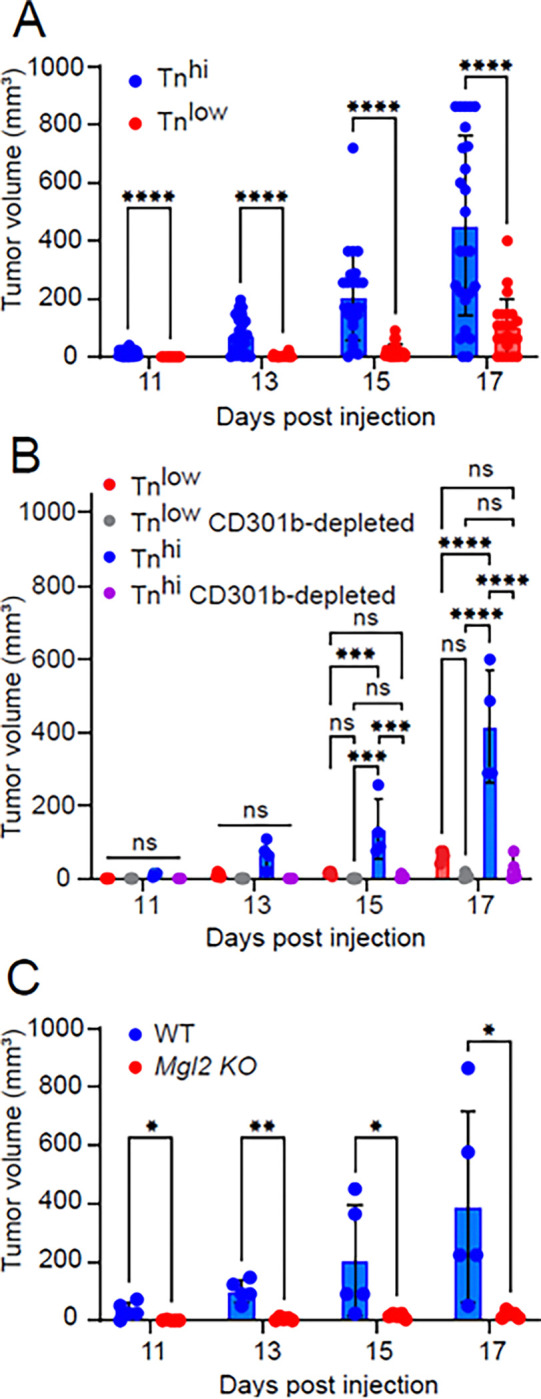
CD301b/Tn Axis Impacts Tumor Growth **A.** AT3 (Tn^low^) and AT3 *Cosmc* KO cells (Tn^hi^) (2.5E5/mouse) were injected into the mammary pads of C57BL/6 mice (n = 25). Tumor sizes were monitored. Tumor sizes were calculated based on (length times width^2^)/2 for volumes in mm^3^. **B.** AT3 (Tn^low^) and AT3 *Cosmc* KO cells (Tn^hi^) tumor cells (2.5×10^5^/mouse) were injected into the mammary pads of WT mice and heterozygous Mgl2-DTR mice (n= 4 or 5) with or without CD301b^+^ cells depleted. Tumor sizes were monitored. **C.** AT3 *Cosmc* KO cells (Tn^hi^) (2.5×10^5^/mouse) were injected into the mammary pads of *Mgl2* KO mice (n=5). Tumor sizes were monitored.

To determine *in vivo* tumor cell growth, we injected AT3 (Tn^lo^) and AT3 *Cosmc* KO (Tn^hi^) murine breast tumor cells into the mammary pads of C57BL/6 mice and monitored tumor growth. We observed that Tn^hi^ tumors grew significantly faster than Tn^lo^ tumors (Fig. 1A), indicating that Tn expression impacts tumor growth rate.

To examine the contribution of CD301b-expressing immune cells to tumor growth, we employed heterozygous *Mgl2*^*+/DTReGFP*^ mice (Mgl2-DTR), in which CD301b+ immune cells can be selectively depleted by diphtheria toxin (DT) treatment ^32,33^. In the first experiment (Fig. 1B), AT3 Tn^low^ or Tn^hi^ tumor cells were injected into Mgl2-DTR mice with or without DT administration. Tumor growth was significantly reduced in DT-treated mice of the Tn^hi^ group, indicating that CD301b^+^ immune cells promote tumor progression. In a complementary experiment (Fig. 1C), we injected AT3 Tn^hi^ cells into homozygous *Mgl2*^*DTReGFP/DTReGFP*^ mice (CD301b-null, *Mgl2* KO), which lack surface expression of CD301b due to the disruption of both alleles but retain the immune cell populations. These mice also exhibited markedly reduced tumor growth compared with wild-type controls. Together, these experiments indicate that the observed phenotype is associated with tumor Tn expression and facilitated by the CD301b protein.

### Tumor-infiltrating CD301b ^+^ cells display a type 2 conventional dendritic cell (cDC2) phenotype.

**Figure 2:**
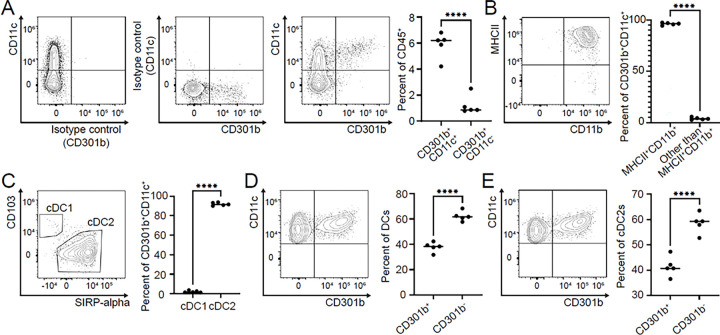
Characterization of CD45^+^CD301b^+^ immune cells in murine breast cancer TME. Single cell suspensions from tumors of WT mice injected with AT3 *Cosmc* KO cells (Tn^hi^) were stained, and expression of surface markers was analyzed with flow cytometry (gated as in Suppl. Fig. 2). **A.** Among live CD45^+^ cells, CD301b^+^ cells are CD11c^+^. **B.** CD301b^+^ and CD11c^+^ cells are MHCII^+^ and CD11b^+^. **C.** CD301b^+^ and CD11c^+^ cells are cDC2. DCs and cDC2s in the TME, **D and E**, respectively, consist of Cd301b-negative and Cd301b-positive cells.

We next characterized tumor-infiltrating CD45^+^CD301b^+^ immune cells in the murine triple-negative breast cancer model. These cells expressed CD11c, a canonical dendritic cell (DC) marker ^42,43^ (Fig. 2A) and were strongly positive for MHCII, confirming their DC identity (Fig. 2B). CD301b^+^ cells also expressed CD11b, a defining marker of mouse type 2 conventional dendritic cells (cDC2s) ^44^ (Fig. 2B). Mouse cDC2s can be distinguished from cDC1s by CD103 and SIRP-alpha expressions ^42,45^. We examined the expression of these markers by tumor-infiltrating CD301b^+^ cells, which display a cDC2 phenotype (Fig. 2C). Although CD301b^+^ cells are primarily cDC2s, not all DCs or cDC2s express CD301b in the TME (Fig. 2D and 2E).

cDC2s constitute a subset of antigen-presenting cells that play key roles in coordinating adaptive immune responses. In contrast to cDC1s, which specialize in cross-presentation and cytotoxic T cell activation, cDC2s promote CD4^+^ T cell priming and modulate immune polarization within tissues ^46–48^. Recent studies have shown that cDC2s exhibit remarkable plasticity in the tumor microenvironment, where they can adopt either immunostimulatory or tolerogenic phenotypes in response to local cues ^48^.

### Tumor-infiltrating CD301b^+^ dendritic cells exhibit enhanced IL-10 expression and remain responsive to TLR4 stimulation

Because CD301b^+^ dendritic cells have been linked to immunoregulatory activity in multiple settings, we next asked whether tumor-infiltrating CD301b^+^ DCs display an IL-10-associated regulatory phenotype. IL-10 is a central anti-inflammatory cytokine that restrains excessive inflammation and suppresses T-cell effector programs through several mechanisms, including inhibition of antigen-presenting cell activation, inflammatory cytokine production, and downstream T-cell priming functions ^49,50^. Prior studies have also associated CD301b/CLEC10A-expressing myeloid cells with immunosuppressive or regulatory functions, including suppression of T follicular helper responses, promotion of metastasis-associated immune suppression, and induction of regulatory programs in CD301b^+^ antigen-presenting cells ^34,51,52^. In addition, tumor-associated glycan-lectin interactions involving the CD301b/CLEC10A axis have been linked to immune modulation in cancer, including breast cancer and Tn-driven tumor models ^35,37^.

To test whether CD301b^+^ DCs in the breast tumor microenvironment (TME) are positioned to contribute to this regulatory axis, we compared IL-10 expression in CD301b^+^ DCs isolated from tumors of wild-type mice under unstimulated conditions and after short-term stimulation with LPS, a TLR4 agonist. Unstimulated CD301b^+^ DCs obtained from the spleens of naïve mice served as the control group. Tumor-derived CD301b^+^ DCs showed a detectable IL-10^+^ expression at baseline, and this population increased further after LPS stimulation, indicating that these cells may contribute to immune regulation within the TME and remain capable of augmenting IL-10 production in response to inflammatory cues. This observation is consistent with prior work showing that CLEC10A/CD301b engagement can cooperate with innate stimuli to enhance IL-10 production in dendritic cells and promote regulatory programming ^51^.

Notably, CD301b^+^ DCs isolated from the TME displayed a greater frequency of IL-10-expressing cells than their splenic counterparts under both unstimulated and LPS-stimulated conditions. These findings suggest that the tumor microenvironment either enriches for an IL-10-competent CD301b^+^ DC population or licenses infiltrating CD301b^+^ DCs toward a more suppressive phenotype after tumor entry. Together, these data support IL-10 production as a functional feature of tumor-associated CD301b^+^ DCs and offer a plausible mechanistic link between CD301b^+^ myeloid cell accumulation and the suppression of productive anti-tumor inflammation. Although these data do not establish IL-10 as the sole mediator of the phenotype, they support IL-10 competence as a functional feature of tumor-associated CD301b^+^ dendritic cells.

### The lack of CD301b is associated with a strong inflammatory immune signature in the breast TME

**Figure 4:**
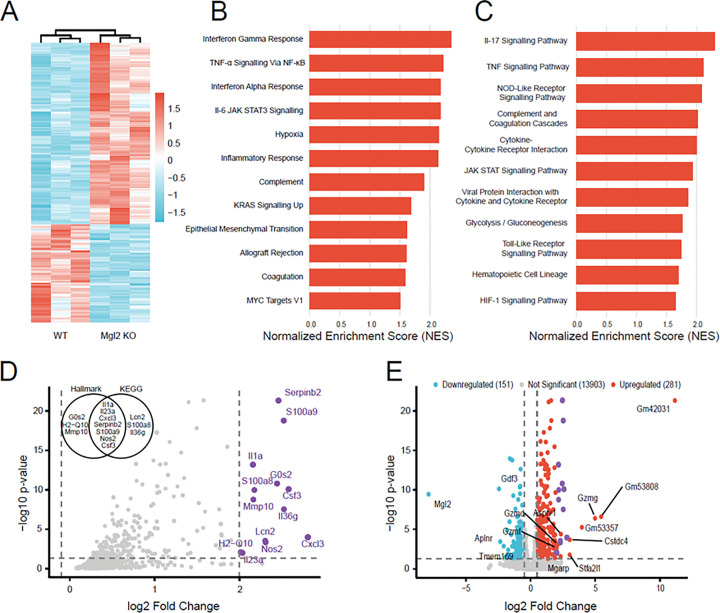
The lack of CD301b is associated with a strong inflammatory immune signature in the breast TME **A.**Heatmap showing differentially expressed genes (DEGs) identified by RNA-seq from tumors in *Mgl2* KO and WT mice. The blue and red bands indicate low and high gene expression quantity, respectively. Biological replicates showed the highest degree of correlation. **B.** Gene Set Enrichment Analysis (GSEA) of Hallmark pathways reveals immune-related and cancer-associated pathways, all of which are upregulated in Mgl2 KO. **C.** GSEA of KEGG pathways shows immune-related and cancer-associated pathways, and they are all upregulated in Mgl2 KO. **D.** Volcano plot showing the pathway-associated genes (from panels B and C). The genes with a log_2_ fold change of 2 or greater are highlighted in purple. The X-axis represents log_2_-transformed fold change, and the Y-axis represents −log_10_-transformed significance. The VENN diagram shows the distribution of genes between Hallmark and KEGG gene sets. **E.**Volcano plot of DEGs between tumors from *Mgl2* KO and WT mice. Red points indicate upregulated DEGs, blue points indicate downregulated DEGs, gray points represent non-significant genes, and purple points indicate the pathway-associated genes (from panel D). All the genes with a log_2_ fold change ≥ 2 are labeled.

Since CD301b directly associates with tumor growth and identifies cDC2 and macrophage populations within the breast tumor microenvironment (TME), we examined how its loss impacts immune signaling and tumor–immune interactions. To this end, we performed bulk RNA sequencing (bulk RNA-seq) on tumors derived from wild-type (WT) and *Mgl2* knockout (*Mgl2* KO) mice following injection of AT3 Tn^hi^ tumor cells. The global transcriptomic heatmap (Fig. 4A) revealed distinct clustering and clear separation between WT and *Mgl2* KO mouse tumors, indicating a strong transcriptional divergence associated with *Mgl2* loss. This observation provided the foundation for downstream pathway and gene-level analyses to elucidate how CD301b influences immune regulation within the TME.

To dissect these transcriptomic differences, Gene Set Enrichment Analysis (GSEA) was performed using the Hallmark and Kyoto Encyclopedia of Genes and Genomes (KEGG) enrichment gene set databases (Fig. 4B–C). *Mgl2* KO tumors displayed significant enrichment of immune and inflammatory pathways, including TNF-α/NF-κB, IL-6/JAK-STAT3, interferon-α/γ, and IL-17 signaling—pathways broadly associated with myeloid activation, cytokine production, and tumor immunosurveillance ^53,54^. Despite the slower tumor growth observed in the knockout mice, complement/coagulation, hypoxia, and glycolytic pathways were also upregulated, suggesting a compensatory increase in metabolic and inflammatory activity within the TME. Collectively, these data suggest that CD301b functions as an immunoregulatory node that tempers cytokine and interferon responses, whereas its loss enhances pro-inflammatory signaling. The dominance of interferon- and NF-κB–driven programs aligns with the slower tumor progression observed in KO mice, implying that CD301b deficiency reprograms the TME toward a functionally immune-active, tumor-controlling state.

The pathway-focused volcano plot (Fig. 4D) highlights key upregulated genes underpinning these responses, including *Cxcl3, Il1a, Il23a, Il36g, Csf3, Nos2, S100a8, S100a9*, and *Lcn2*. These represent canonical NF-κB and IL-17 targets known to mediate myeloid recruitment, nitric oxide production, and acute-phase inflammation—hallmarks of innate immune activation ^55–57^. The upregulation of the serine protease inhibitor and matrix metalloproteinase genes *Serpinb2* and *Mmp10* in the tumor microenvironment of knockout mice suggests a tumor-suppressive function through the regulation of extracellular matrix remodeling and modulation of immune responses ^58,59^. Thus, CD301b loss is associated with a more inflammatory transcriptional profile in the tumor microenvironment, characterized by innate immune activation, interferon-associated programs, and concurrent tissue-remodeling features.

The volcano plot of all differentially expressed genes (DEGs) (Fig. 4E) contextualizes these changes within the full transcriptome. Upregulated genes largely mirrored those driving enriched pathways, confirming that *Cxcl3, Il1a, Il23a, Nos2, S100a8/a9*, and *Csf3* constitute the core *Mgl2* KO transcriptional program rather than isolated pathway artifacts. On the other hand, downregulation of the TGF-β family growth differentiation factor 3 and the apelin receptor genes *Gdf3* and *Aplnr* may be relevant to pathways involved in tumor growth, angiogenesis, and metastasis ^60,61^.

Together, these results suggest that CD301b deficiency reprograms the TME toward an inflammatory and interferon-dominant transcriptional state associated with delayed tumor growth. These findings support the interpretation that CD301b functions as an immunoregulatory myeloid node whose loss releases inflammatory immune programs within the breast tumor microenvironment.

### CLEC10A^+^ myeloid cells in the human breast cancer TME include both dendritic cells and macrophages

**Figure 5:**
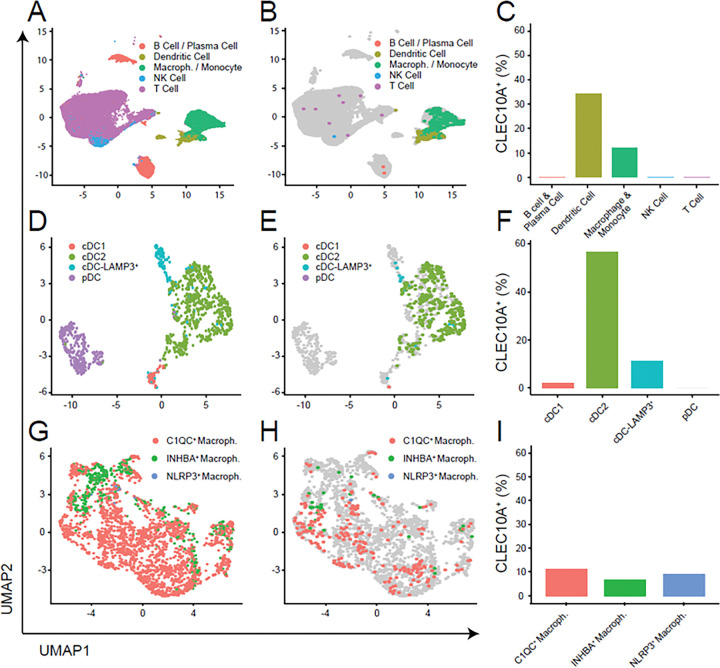
Characterization of CD45^+^CLEC10A^+^ myeloid cells in human breast cancer TME. **A.** UMAP of all immune cells colored by major lineage (B/Plasma cell, Dendritic cell, Macrophage/Monocyte, NK cell, T cell). **B.** Immune cells highlighting CLEC10A^+^ cells (colored) over all cells (gray). **C.** Bar plot showing the fraction of CLEC10A^+^ cells within each major lineage. **D.** UMAP of dendritic cell compartment colored by subset (cDC1, cDC2, cDC-LAMP3^+^, pDC). **E.** Dendritic cell subset highlighting CLEC10A^+^ cells (colored) over all DCs (gray). **F.** Bar plot showing the fraction of CLEC10A^+^ cells within each DC subset. **G.** UMAP of macrophage compartment colored by subset: C1QC^+^ macrophages, INHBA^+^ macrophages, and NLRP3^+^ macrophages. **H.** Macrophage subset highlighting CLEC10A^+^ cells (colored) over all macrophages (gray). **I.** Bar plot showing the fraction of CLEC10A^+^ cells within each macrophage subset. UMAP axes indicate the first two dimensions. Percentages in bar plots are calculated as (CLEC10A^+^ cells / total cells) within the indicated group.

To determine how CLEC10A maps onto human breast tumor myeloid populations, we next analyzed publicly available single-cell RNA sequencing (scRNA-seq) data (GSE161529) from 20 patients, including triple-negative (n = 8), ER^+^ (n = 6), and HER2^+^ (n = 6) tumors ^62^. Following quality control, data integration, and annotation, we focused on CD45^+^ immune cells to map CLEC10A expression across myeloid populations (Fig. 5A; Suppl. Figure 3A). CLEC10A expression was most prominent in dendritic cells and was also detectable in macrophage/monocyte populations, with negligible expression in other immune cells (Fig. 5B–C). Across breast cancer subtypes, dendritic cells consistently showed higher CLEC10A expression than macrophages (Suppl. Figure 3B). Within the dendritic cell compartment, we identified four subsets—cDC1, cDC2, cDC-LAMP3^+^, and plasmacytoid DC (pDC)—and found that CLEC10A expression was highest in cDC2 (56.9%) and moderate in cDC-LAMP3^+^ (11.3%), but low in cDC1 (1.9%) and absent in pDCs (0%) (Fig. 5D–F; Suppl. Figure 3C–D).

We next examined tumor-associated macrophages (TAMs). TAMs are key regulators of tumor inflammation, tissue remodeling, and immune suppression ^63^. TAMs were subdivided into transcriptionally defined subsets reflecting distinct functional programs: C1QC^+^ macrophages, associated with immunosuppression ^64–66^; NLRP3^+^ macrophages, associated with poor prognosis and tumor growth ^67,68^; and INHBA^+^ macrophages, linked to angiogenesis, matrix remodeling, and tumor progression ^69^ (Fig. 5G; Suppl. Figure 3E). Among these subsets, *CLEC10A* transcription distributed similarly in C1QC^+^ macrophages (11%), NLRP3^+^ macrophages (9%), and INHBA^+^ macrophages (7%) (Fig. 5H–I). Interestingly, CLEC10A-positive NLRP3^+^ macrophages were detected exclusively in triple-negative breast cancers, where expression levels were comparable to those in INHBA^+^ macrophages (Suppl. Figure 3F). This enrichment suggests that CLEC10A expression extends beyond dendritic cells to macrophage populations, particularly those engaged in inflammatory and tissue-remodeling responses within aggressive tumor subtypes.

Taken together, these results indicate that CLEC10A expression in the human breast cancer microenvironment is concentrated within myeloid lineages—encompassing both cDC2-like dendritic cells and specialized macrophage subsets. This pattern mirrors the cellular distribution observed in the mouse TME, suggesting that CLEC10A/CD301b marks a conserved myeloid program potentially involved in coordinating immune regulation and tissue remodeling during tumor progression.

### CLEC10A marks a convergent transcriptional state across human breast cancer cDC2 and macrophages

**Figure 6:**
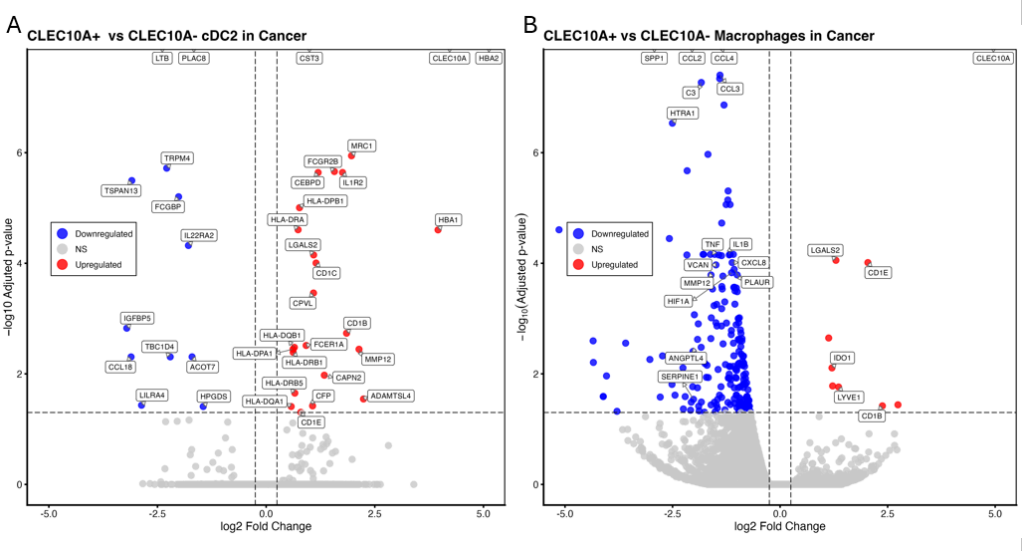
Differential gene expression associated with CLEC10A expression in human breast cancer cDC2 and macrophages. **A.** Volcano plot of differential gene expression between CLEC10A^−^ and CLEC10A^+^ cDC2 from the scRNA-seq dataset shown in Fig. 5. cDC2 from all human breast cancer samples were subsetted, pseudobulk counts were generated per patient, and differential expression was assessed using edgeR. **B.** Volcano plot of differential gene expression between CLEC10A^−^ and CLEC10A^+^ macrophages from the same scRNA-seq dataset. Macrophages were subsetted, pseudobulk counts were generated per patient, and differential expression was assessed using edgeR.

To define the transcriptional programs associated with CLEC10A expression in human breast tumor myeloid cells, we performed differential expression analysis on a per-patient basis comparing CLEC10A^+^ and CLEC10A^−^ cells within the cDC2 and macrophage compartments from all human breast cancer samples shown in Fig. 5 (Fig. 6A–B). In cDC2, CLEC10A^+^ cells were enriched for canonical antigen-presentation and cDC2-associated genes, including CLEC10A, CD1C, CD1B, FCER1A, and multiple HLA class II genes, confirming that CLEC10A identifies a differentiated antigen-presenting cDC2 state. CLEC10A^+^ cDC2 also upregulated IL1R2, FCGR2B, MRC1, and MMP12, consistent with an immune-regulatory and tumor-conditioning program. In contrast, CLEC10A^−^ cDC2 showed relatively higher expression of genes such as LTB, PLAC8, CST3, FCGBP, and IL22RA2, indicating a transcriptionally distinct state with less canonical antigen-presentation identity and greater inflammatory or tissue-reactive features.

A parallel pattern emerged in macrophages. CLEC10A^+^ macrophages upregulated CLEC10A together with CD1E, CD1B, LGALS2, IDO1, and LYVE1, supporting a state linked to antigen handling, immune regulation, and tissue-remodeling functions. By contrast, CLEC10A^−^ macrophages preferentially expressed inflammatory mediators and monocyte/TAM-associated genes, including SPP1, CCL2, CCL3, CCL4, TNF, IL1B, CXCL8, VCAN, PLAUR, HIF1A, ANGPTL4, and SERPINE1, consistent with a more inflammatory and tumor-promoting phenotype. Thus, the human cDC2 and macrophage datasets are concordant in showing that CLEC10A marks a shared myeloid state characterized by stronger antigen-presentation features but restrained inflammatory cytokine and chemokine programs. This interpretation is consistent with the mouse data: CD301b prominently marked cDC2s in tumors, whereas in vivo loss of CD301b was associated with a broad induction of inflammatory pathways and innate cytokine programs, as inferred from bulk RNA-seq. Together, the human single-cell and mouse bulk RNA-seq data support a model in which CLEC10A/CD301b identifies a conserved myeloid program associated with stronger antigen-presentation features and reduced inflammatory cytokine and chemokine expression, consistent with a more regulatory state in the breast tumor microenvironment.

## Discussion

Aberrant glycosylation is a hallmark of malignant transformation, and our findings identify CD301b as a key immunoregulatory lectin that links breast tumor-associated Tn antigens to myeloid immune modulation. CD301b^+^ cells, primarily cDC2s, promoted breast tumor growth, whereas their depletion or genetic loss limited progression. These observations align with previous studies demonstrating that TACAs interact with lectins to influence myeloid differentiation and immune regulation ^19,21,22,70–72^. In breast cancer, the Tn-CD301b/CLEC10A axis can now be traced across tumor glycosylation, murine in vivo tumor growth, tumor transcriptomic reprogramming, and human myeloid single-cell states.

Transcriptomic profiling of tumors developed in *Mgl2*-KO mice revealed broad activation of NF-κB, IL-6–JAK–STAT3, and interferon pathways, consistent with a shift toward a more inflammatory transcriptional state within the TME ^73^. These data position CD301b as an immunoregulatory node that tempers innate activation, similar in concept to a checkpoint-like mechanism operating within the myeloid compartment ^9,10,74,75^. While this study does not define the signaling circuitry involved, it indicates that CD301b-expressing myeloid populations, particularly cDC2s and potentially macrophages, contribute to an immunoregulatory phenotype in the breast cancer TME. In support of this interpretation, tumor-infiltrating CD301b^+^ dendritic cells displayed higher IL-10 expression than splenic CD301b^+^ DCs at baseline and after LPS stimulation, indicating that the tumor microenvironment enriches for, or licenses, a CD301b^+^ DC population with enhanced regulatory cytokine competence.

The complex and pleiotropic nature of CD301b’s activity may explain its influence on the immune landscape of cancer ^74–76^. CD301b’s modulatory behavior mirrors that of other C-type lectin receptors ^77^. CD301b-mediated restraint may protect against chronic inflammation, but in tumors, it can inadvertently favor immune escape ^78,79^. Conversely, CD301b loss triggers NF-κB- and interferon-associated inflammation, while also imposing metabolic and hypoxic stress on the TME^80^. Together, these findings support a model in which CD301b functions as a molecular regulator balancing immune activation and tolerance in breast cancer.

A growing mechanistic literature contextualizes the downstream programming capacity of the CLEC10A/CD301b lectin axis. In human antigen-presenting cells, CLEC10A engagement has been repeatedly linked to regulatory polarization, often characterized by enhanced IL-10 production and consequent dampening or redirection of effector T-cell responses^37,51^. Importantly for mouse interpretation, CD301b pathways have been linked to IL-10 induction across multiple experimental systems, indicating that IL-10 skewing is a conserved output that should be integrated into models of CD301b-driven immune modulation^35,51^. A recent ligand-centric study further shows that distinct GalNAc-containing ligands can tune CLEC10A signaling strength and dendritic cell transcriptional outputs, supporting the idea that qualitative features of tumor-associated glycan ligands may shift the balance between inflammatory and regulatory programs in the TME ^81^. Our findings are consistent with this concept at both functional and transcriptional levels: in mice, tumor-associated CD301b^+^ DCs exhibited enhanced IL-10 competence, whereas in human breast cancer, CLEC10A^+^ cDC2s and macrophages displayed a distinct transcriptional state enriched for canonical cDC2 genes and genes associated with immune dampening and tumor conditioning.

Therefore, our findings support CD301b-Tn interactions as an immune-regulatory axis in the breast TME that may regulate antigen processing, cytokine release, or costimulatory signaling within dendritic cells, thereby shaping T cell activation thresholds ^77^. These possibilities highlight the clinical potential of CD301b as a new immune-regulatory axis distinct from canonical checkpoints, such as PD-1 or CTLA-4. A limitation of the current study is that the downstream effects of this myeloid program on lymphocytes remain unresolved. Thus, while our data support a primary role for CD301b-associated myeloid reprogramming, they do not yet define how this state influences T-cell or NK-cell recruitment, activation, spatial organization, or effector function in the tumor microenvironment. Future studies will be needed to determine whether altered cytokine or chemokine production, antigen presentation, or other indirect mechanisms link CD301b-dependent myeloid states to downstream antitumor immunity. A key next step will be to define how specific TACA ligand structures and intracellular adaptors determine signaling strength, pathway selection, and the balance between immunoregulatory signaling, cytokine outputs, and anti-tumor effector programs in vivo. Further single-cell and spatial analyses of the breast TME transcriptome, with and without CD301b, will help define how CD301b^+^ subsets integrate into existing immune networks across tumor stages.

From a translational perspective, targeting the CLEC10A/CD301b-Tn interaction offers a promising route to modulate the breast cancer immune microenvironment. Pharmacologic blockade or glycomimetic interference could complement checkpoint inhibitors by dismantling glycan-mediated myeloid suppression, whereas selective induction of this pathway may have therapeutic relevance in autoimmune disease ^82^. Ultimately, elucidating the intricate mechanisms that govern immune modulation through CD301b will be essential to selectively induce these properties in disease-specific contexts, enabling the development of knowledge-based, precision immunotherapies.

In summary, this study identifies an immunomodulatory CD301b^+^ myeloid phenotype that contributes to breast cancer growth and whose loss induces a robust inflammatory program in the breast TME. Our results further suggest that this phenotype includes tumor-associated CD301b^+^ dendritic cells with enhanced IL-10 expression and conserved human CLEC10A^+^ cDC2 and macrophage transcriptional states linked to immune dampening and tumor conditioning. While the molecular mechanisms remain to be defined, our data indicate that CD301b acts as a glycan-sensitive, checkpoint-like regulator of myeloid activity. Elucidating its ligand specificity and downstream signaling will clarify how breast tumor glycosylation reshapes the TME and may reveal new strategies to enhance immunotherapy efficacy.

## Materials and Methods

### Mice

Eight-week-old female C57BL/6 mice were obtained from Jackson Laboratories (Bar Harbor, ME) and housed at Emory University Whitehead Biomedical Research Building. *Mgl2*^*DTReGFP/DTReGFP*^ mice were a generous gift from Akiko Iwasaki at Yale University. To obtain heterozygous *Mgl2*^*+/DTReGFP*^ mice, C57BL/6 were bred with *Mgl2*^*DTReGFP/DTReGFP*^ mice. Mice were kept in microisolator cages and handled under biosafety level 2 (BSL2) hoods. For tissue processing and subsequent flow cytometry, mice were euthanized by carbon dioxide inhalation in accordance with IACUC guidelines. Where applicable, cell suspensions were generated through mechanical tissue disruption and collagenase D digestion. Red blood cells were lysed, and samples were filtered through 60 μm nylon filters to obtain single-cell suspensions. For depletion of CD301b^+^ cells, heterozygous Mgl2-DTR mice were treated with diphtheria toxin (0.5μg/mouse) in sterile PBS intraperitoneally every two to three days, starting at day − 1 before tumor injection.

All mouse experiments were in compliance with the Emory University Institutional Animal Care and Use Committee under an approved animal use protocol. Our animal use protocol adheres to the principles outlined in *U.S. Government Principles for the Utilization and Care of Vertebrate Animals Used in Testing*, *Research and Training*, the Animal Welfare Act, the *Guide for the Care and Use of Laboratory Animals*, and the *AVMA Guidelines for the Euthanasia of Animals*.

### Generation of Tn breast cancer cells

To express Tn glycans at high levels in tumor cells, we used a CRISPR/Cas9 methodology to stably silence Cosmc expression in AT3 cells using established protocols and reagents. Mouse *Cosmc* guide RNA and CRISPR/Cas9 plasmid were obtained from Santa Cruz Technology (sc-425587). AT3 murine breast cancer cells were a generous gift from the Kebin Liu lab at Augusta University. AT3 cells were transfected with *Cosmc* CRISPR/Cas9 KO plasmid according to the manufacturer’s protocol. Puromycin was used to select transfected cells. We then used flow cytometry to confirm higher expression of Tn on the AT3 cell surfaces using the ReBaG6 antibody (generously provided by Richard Cummings at Harvard University) (Suppl. Figure 1A) ^41^ and VVL lectin (Vector Laboratories) (Suppl. Figure 1B). The proliferations of transfected and untransfected cell lines (AT3 Tn^hi^ and AT3 Tn^low^) were tested in an MTT proliferation assay for three days, and colorimetric analysis was performed with a CytoTek plate reader according to protocol; no significant differences in proliferation compared to parent cells were observed (Suppl. Figure 1D). Cell lines were maintained in RPMI media supplemented with 10% FBS, sodium pyruvate, HEPES buffer, NEAA, β-mercaptoethanol, and penicillin/streptomycin at 37°C, 5% CO2.

### AT3 Tn and/or AT3 Tn Tumor Challenge

AT3 cells were harvested and washed in sterile PBS. Cells were suspended in a final concentration of 2.5E6/ml sterile PBS. Cells were subcutaneously injected into the mammary pads of mice at 2.5E5/100μl/mouse. Mice were monitored throughout the experiment and euthanized at the tumor endpoint, defined as the maximum tumor dimension of 0.9–1.2 cm. Tumor volumes were calculated as ((length × width × width)/2) in mm^3^.

### Flow Cytometry

Cells were stained in PBS with TruStain fcX (BioLegend, Cat. No. 101320) to reduce non-specific antibody binding. Cell samples were stained with the following antibodies and stains: CD11c-PacBlue (BioLegend, clone N418), CD11b-Alexa Fluor 488 (BioLegend, clone M1/70), CD11b-BUV 805 (Invitrogen, clone M1/70), SIRPα-Alexa Fluor 700 (BioLegend, clone P84), CD103-PE/Dazzle 594 (BioLegend, clone 2E7), MHCII-BV785 (BioLegend, clone M5/114.15.2), CD45-Spark UV 387 (BioLegend, clone 30-F11), CD45-Alexa Fluor 647 (BioLegend, clone 30-F11), IL-10-APC (BioLegend, clone JES5–16E3), CD301b-PE (BioLegend, clone URA-1), and LIVE/DEAD Fixable Blue (Invitrogen). All isotype controls were obtained from BioLegend. Samples were washed and analyzed with flow cytometry (Cytek Aurora). Fluorescence minus one (FMO) plus specific isotype control antibody-stained samples were used as negative staining controls, and single stains were used for compensation. Flow cytometry data were analyzed using FlowJo Single Cell Analysis Software. For IL-10 staining, cells were incubated for 4 hours in the presence of GolgiStop and GolgiPlug. The concentration of LPS used for *in vitro* stimulation was 1 μg/ml.

### Bulk RNA-seq and Bioinformatics

Tumors were harvested from wild-type C57BL/6J and *Mgl2* KO mice (n = 3 per group) at the experimental endpoint defined in the tumor challenge protocol. Total RNA was extracted using the MagMAX^™^ mirVana^™^ Total RNA Isolation Kit in combination with the KingFisher Apex system (Thermo Fisher Scientific). RNA integrity and concentration were assessed with a Qubit 3.0 fluorometer (Thermo Fisher Scientific). High-quality RNA samples were used for library preparation, followed by quality control and sequencing using Novogene’s standard protocol. Libraries were sequenced on the Illumina NovaSeq X Plus platform to generate paired-end 150 bp reads (PE150) at Novogene Inc. Raw FASTQ files were retrieved and subjected to quality control with FastQC ^83^. Reads were aligned to the *Mus musculus* reference genome (GRCm39/mm39) using HISAT2 ^84^. A gene-level count matrix was generated with featureCounts ^85^. The count matrix was imported to the downstream differential expression analysis using the *DESeq2* R package ^86^. Significantly differentially expressed genes were defined by adjusted p-value < 0.05. Gene Set Enrichment Analysis (GSEA) was performed on the RNA-seq dataset using the clusterProfiler R package ^87^ to identify significantly enriched KEGG and hallmark pathways between *Mgl2* KO and wild-type tumors. To focus on cancer-relevant biology, enrichment results were refined to include immune- and cancer-related pathways.

### Analyzing the scRNA-seq data

scRNA-seq data generated using the 10X Genomics Chromium platform were obtained from the Gene Expression Omnibus (GEO; accession GSE161529) ^88^. Data processing was performed using the Seurat R package (version 5). Low-quality cells were excluded based on standard Seurat quality-control metrics, ^89^ and putative doublets were removed using DoubletFinder. ^90^ The datasets were integrated using the Seurat reciprocal PCA (RPCA)-based integration workflow. Cell-type annotation was subsequently performed using scATOMIC, ^91^ and dendritic cell and macrophage subsets were further confirmed using canonical lineage markers from CellMarker 2.0.^92,93^

For differential expression analysis, cDC2 and macrophage populations were analyzed separately by generating donor-level pseudobulk profiles, with CLEC10A-positive and CLEC10A-negative cells defined as the comparison groups within each cell type. Differential expression testing was then performed using edgeR ^94^ with a design matrix including group and donor as covariates, thereby controlling for inter-donor variability.

### Statistical Analysis

GraphPad Prism v8 was used for statistical analyses. Two-way ANOVA with Tukey’s multiple comparisons test was used to determine statistical significance between experimental groups in each of the applicable experimental models (Fig. 1B). An unpaired parametric two-tailed t-test was used for Fig. 1A, 1C, 2A, 2B, 2C, 2D, 2E, and Suppl. Figure 1C. Significance is indicated on each graph based on p-value: >0.05 = ns; <.05 = *; <0.01 = **; <0.001 = ***; <0.0001 = ****.

## Supplementary Material

Supplementary Files

This is a list of supplementary files associated with this preprint. Click to download.

• NPJBCsupplfigures0406.pptx

## Figures and Tables

**Figure 3:**
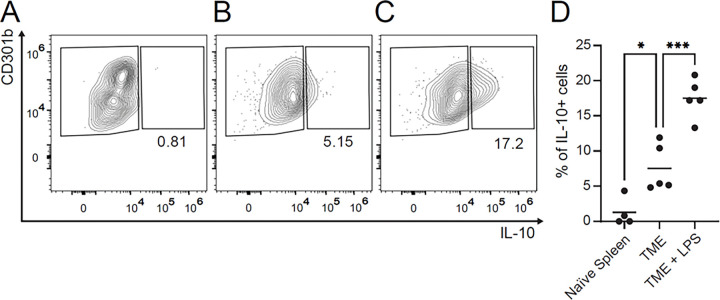
CD301b+ dendritic cells in the tumor microenvironment express elevated IL-10 and remain responsive to LPS stimulation. CD301b+ DCs isolated from tumors showed a greater frequency of IL-10-expressing cells than CD301b+ DCs from naïve spleens. LPS increased the proportion of IL-10+ cells in TME DCs.

## References

[1] Zhang Y. & Zhang Z. The history and advances in cancer immunotherapy: understanding the characteristics of tumor-infiltrating immune cells and their therapeutic implications. Cell Mol Immunol 17, 807–821 (2020). 10.1038/s41423-020-0488-6 32612154 PMC7395159

[2] Swoboda A. & Nanda R. Immune Checkpoint Blockade for Breast Cancer. Cancer Treat Res 173, 155–165 (2018). 10.1007/978-3-319-70197-4_10 29349763 PMC6061922

[3] Fiorentino D. F., Bond M. W. & Mosmann T. R. Two types of mouse T helper cell. IV. Th2 clones secrete a factor that inhibits cytokine production by Th1 clones. J Exp Med 170, 2081–2095 (1989). 10.1084/jem.170.6.2081 2531194 PMC2189521

[4] Acuner Ozbabacan S. E., Gursoy A., Nussinov R. & Keskin O. The structural pathway of interleukin 1 (IL-1) initiated signaling reveals mechanisms of oncogenic mutations and SNPs in inflammation and cancer. PLoS Comput Biol 10, e1003470 (2014). 10.1371/journal.pcbi.1003470 24550720 PMC3923659

[5] Freeman G. J. Engagement of the PD-1 immunoinhibitory receptor by a novel B7 family member leads to negative regulation of lymphocyte activation. J Exp Med 192, 1027–1034 (2000). 11015443 10.1084/jem.192.7.1027 PMC2193311

[6] Rodrigues Mantuano N., Natoli M., Zippelius A. & Laubli H. Tumor-associated carbohydrates and immunomodulatory lectins as targets for cancer immunotherapy. J Immunother Cancer 8 (2020). 10.1136/jitc-2020-001222 PMC7537339 33020245

[7] Laubli H. & Varki A. Sialic acid-binding immunoglobulin-like lectins (Siglecs) detect self-associated molecular patterns to regulate immune responses. Cell Mol Life Sci 77, 593–605 (2020). 10.1007/s00018-019-03288-x 31485715 PMC7942692

[8] Pinho S. S. & Reis C. A. Glycosylation in cancer: mechanisms and clinical implications. Nat Rev Cancer 15, 540–555 (2015). 10.1038/nrc3982 26289314

[9] Marino K. V., Cagnoni A. J., Croci D. O. & Rabinovich G. A. Targeting galectin-driven regulatory circuits in cancer and fibrosis. Nat Rev Drug Discov 22, 295–316 (2023). 10.1038/s41573-023-00636-2 36759557

[10] Cagnoni A. J. Galectin-1 fosters an immunosuppressive microenvironment in colorectal cancer by reprogramming CD8(+) regulatory T cells. Proc Natl Acad Sci U S A 118 (2021). 10.1073/pnas.2102950118 PMC8166155 34006646

[11] Paschall A. V. M., D. R.; Avci F. Y. in Encyclopedia of Cell Biology, Second Edition (ed R. A.; Hart Bradshaw G. W; Stahl P. D.) 404–413 (Elsevier, 2023).

[12] Beatson R. The mucin MUC1 modulates the tumor immunological microenvironment through engagement of the lectin Siglec-9. Nat Immunol 17, 1273–1281 (2016). 10.1038/ni.3552 27595232 PMC5257269

[13] Marcos N. T. Role of the human ST6GalNAc-I and ST6GalNAc-II in the synthesis of the cancer-associated sialyl-Tn antigen. Cancer Res 64, 7050–7057 (2004). 10.1158/0008-5472.CAN-04-1921 15466199

[14] Schultz M. J. The Tumor-Associated Glycosyltransferase ST6Gal-I Regulates Stem Cell Transcription Factors and Confers a Cancer Stem Cell Phenotype. Cancer Res 76, 3978–3988 (2016). 10.1158/0008-5472.CAN-15-2834 27216178 PMC4930726

[15] Freire T., Lo-Man R., Bay S. & Leclerc C. Tn glycosylation of the MUC6 protein modulates its immunogenicity and promotes the induction of Th17-biased T cell responses. J Biol Chem 286, 7797–7811 (2011). 10.1074/jbc.M110.209742 21193402 PMC3048667

[16] Saeland E. The C-type lectin MGL expressed by dendritic cells detects glycan changes on MUC1 in colon carcinoma. Cancer Immunol Immunother 56, 1225–1236 (2007). 10.1007/s00262-006-0274-z 17195076 PMC11031027

[17] van Vliet S. J., Gringhuis S. I., Geijtenbeek T. B. & van Kooyk Y. Regulation of effector T cells by antigen-presenting cells via interaction of the C-type lectin MGL with CD45. Nat Immunol 7, 1200–1208 (2006). 10.1038/ni1390 16998493

[18] Cheever M. A. The prioritization of cancer antigens: a national cancer institute pilot project for the acceleration of translational research. Clin Cancer Res 15, 5323–5337 (2009). 10.1158/1078-0432.CCR-09-0737 19723653 PMC5779623

[19] Tumoglu B., Keelaghan A. & Avci F. Y. Tn antigen interactions of macrophage galactose-type lectin (MGL) in immune function and disease. Glycobiology 33, 879–887 (2023). 10.1093/glycob/cwad083 37847609 PMC10859631

[20] Wang Y. Cosmc is an essential chaperone for correct protein O-glycosylation. Proc Natl Acad Sci U S A 107, 9228–9233 (2010). 10.1073/pnas.0914004107 20439703 PMC2889116

[21] Ju T., Aryal R. P., Kudelka M. R., Wang Y. & Cummings R. D. The Cosmc connection to the Tn antigen in cancer. Cancer Biomark 14, 63–81 (2014). 10.3233/CBM-130375 24643043 PMC5808877

[22] Beatson R. The Breast Cancer-Associated Glycoforms of MUC1, MUC1-Tn and sialyl-Tn, Are Expressed in COSMC Wild-Type Cells and Bind the C-Type Lectin MGL. PLoS One 10, e0125994 (2015). 10.1371/journal.pone.0125994 25951175 PMC4423978

[23] Beatson R. E., Taylor-Papadimitriou J. & Burchell J. M. MUC1 immunotherapy. Immunotherapy 2, 305–327 (2010). 10.2217/imt.10.17 20635898

[24] Siroy A. MUC1 is expressed at high frequency in early-stage basal-like triple-negative breast cancer. Hum Pathol 44, 2159–2166 (2013). 10.1016/j.humpath.2013.04.010 23845471 PMC4167755

[25] Burchell J. M., Beatson R., Graham R., Taylor-Papadimitriou J. & Tajadura-Ortega V. O-linked mucin-type glycosylation in breast cancer. Biochem Soc Trans 46, 779–788 (2018). 10.1042/BST20170483 29903935 PMC6103458

[26] Chen D. Immunotherapy of spontaneous mammary carcinoma with fusions of dendritic cells and mucin 1-positive carcinoma cells. Immunology 109, 300–307 (2003). 12757626 10.1046/j.1365-2567.2003.01656.x PMC1782954

[27] Mukherjee P. MUC1-specific immune therapy generates a strong anti-tumor response in a MUC1-tolerant colon cancer model. Vaccine 25, 1607–1618 (2007). 10.1016/j.vaccine.2006.11.007 17166639 PMC1810513

[28] Lakshminarayanan V. Immune recognition of tumor-associated mucin MUC1 is achieved by a fully synthetic aberrantly glycosylated MUC1 tripartite vaccine. Proc Natl Acad Sci U S A 109, 261–266 (2012). 10.1073/pnas.1115166109 22171012 PMC3252914

[29] Loureiro L. R. Challenges in Antibody Development against Tn and Sialyl-Tn Antigens. Biomolecules 5, 1783–1809 (2015). 10.3390/biom5031783 26270678 PMC4598775

[30] Rodriguez E. Fasciola hepatica Immune Regulates CD11c+ Cells by Interacting with the Macrophage Gal/GalNAc Lectin. Front Immunol 8, 264 (2017). 10.3389/fimmu.2017.00264 28360908 PMC5350155

[31] Singh S. K. Characterization of murine MGL1 and MGL2 C-type lectins: distinct glycan specificities and tumor binding properties. Mol Immunol 46, 1240–1249 (2009). 10.1016/j.molimm.2008.11.021 19162326

[32] Kumamoto Y., Hirai T., Wong P. W., Kaplan D. H. & Iwasaki A. CD301b+ dendritic cells suppress T follicular helper cells and antibody responses to protein antigens. Elife 5 (2016). 10.7554/eLife.17979 PMC5033605 27657168

[33] Kumamoto Y. CD301b(+) dermal dendritic cells drive T helper 2 cell-mediated immunity. Immunity 39, 733–743 (2013). 10.1016/j.immuni.2013.08.029 24076051 PMC3819035

[34] Kenkel J. A. An Immunosuppressive Dendritic Cell Subset Accumulates at Secondary Sites and Promotes Metastasis in Pancreatic Cancer. Cancer Res 77, 4158–4170 (2017). 10.1158/0008-5472.CAN-16-2212 28611041 PMC5550516

[35] da Costa V. The Tn antigen promotes lung tumor growth by fostering immunosuppression and angiogenesis via interaction with Macrophage Galactose-type lectin 2 (MGL2). Cancer Lett 518, 72–81 (2021). 10.1016/j.canlet.2021.06.012 34144098

[36] Kurze A. K. Immature O-glycans recognized by the macrophage glycoreceptor CLEC10A (MGL) are induced by 4-hydroxy-tamoxifen, oxidative stress and DNA-damage in breast cancer cells. Cell Commun Signal 17, 107 (2019). 10.1186/s12964-019-0420-9 31455323 PMC6712659

[37] Wegscheider A. S. CD301 and LSECtin glycan-binding receptors of innate immune cells serve as prognostic markers and potential predictors of immune response in breast cancer subtypes. Glycobiology 34 (2024). 10.1093/glycob/cwae003 PMC10987291 38206856

[38] Tang S. CLEC10A can serve as a potential therapeutic target and its level correlates with immune infiltration in breast cancer. Oncol Lett 24, 285 (2022). 10.3892/ol.2022.13405 35814828 PMC9260715

[39] Qin Y. Immunological role and prognostic potential of CLEC10A in pan-cancer. Am J Transl Res 14, 2844–2860 (2022). 35702069 PMC9185031

[40] Ju T. & Cummings R. D. A unique molecular chaperone Cosmc required for activity of the mammalian core 1 beta 3-galactosyltransferase. Proc Natl Acad Sci U S A 99, 16613–16618 (2002). 10.1073/pnas.262438199 12464682 PMC139192

[41] Matsumoto Y. Identification of Tn antigen O-GalNAc-expressing glycoproteins in human carcinomas using novel anti-Tn recombinant antibodies. Glycobiology 30, 282–300 (2020). 10.1093/glycob/cwz095 31742337 PMC7175968

[42] Breed E. R. Type 2 cytokines in the thymus activate Sirpalpha(+) dendritic cells to promote clonal deletion. Nat Immunol 23, 1042–1051 (2022). 10.1038/s41590-022-01218-x 35637352 PMC10037932

[43] Kumamoto Y., Denda-Nagai K., Aida S., Higashi N. & Irimura T. MGL2 Dermal dendritic cells are sufficient to initiate contact hypersensitivity in vivo. PLoS One 4, e5619 (2009). 10.1371/journal.pone.0005619 19440334 PMC2680031

[44] Izumi G. CD11b(+) lung dendritic cells at different stages of maturation induce Th17 or Th2 differentiation. Nat Commun 12, 5029 (2021). 10.1038/s41467-021-25307-x 34413303 PMC8377117

[45] Bottcher J. P. & Reis e Sousa C. The Role of Type 1 Conventional Dendritic Cells in Cancer Immunity. Trends Cancer 4, 784–792 (2018). 10.1016/j.trecan.2018.09.001 30352680 PMC6207145

[46] Haniffa M. Human tissues contain CD141hi cross-presenting dendritic cells with functional homology to mouse CD103+ nonlymphoid dendritic cells. Immunity 37, 60–73 (2012). 10.1016/j.immuni.2012.04.012 22795876 PMC3476529

[47] Liu P., Zhao L., Kroemer G. & Kepp O. Conventional type 1 dendritic cells (cDC1) in cancer immunity. Biol Direct 18, 71 (2023). 10.1186/s13062-023-00430-5 37907944 PMC10619282

[48] Saito Y., Komori S., Kotani T., Murata Y. & Matozaki T. The Role of Type-2 Conventional Dendritic Cells in the Regulation of Tumor Immunity. Cancers (Basel) 14 (2022). 10.3390/cancers14081976 PMC9028336 35454882

[49] Akdis C. A. & Blaser K. Mechanisms of interleukin-10-mediated immune suppression. Immunology 103, 131–136 (2001). 10.1046/j.1365-2567.2001.01235.x 11412299 PMC1783236

[50] Carlini V. The multifaceted nature of IL-10: regulation, role in immunological homeostasis and its relevance to cancer, COVID-19 and post-COVID conditions. Front Immunol 14, 1161067 (2023). 10.3389/fimmu.2023.1161067 37359549 PMC10287165

[51] Costa M. Macrophage Gal/GalNAc lectin 2 (MGL2)(+) peritoneal antigen presenting cells during Fasciola hepatica infection are essential for regulatory T cell induction. Sci Rep 12, 17661 (2022). 10.1038/s41598-022-21520-w 36271272 PMC9587262

[52] Kumamoto Y., Hirai T., Wong P. W., Kaplan D. H. & Iwasaki A. CD301b(+) dendritic cells suppress T follicular helper cells and antibody responses to protein antigens. Elife 5 (2016). 10.7554/eLife.17979 PMC5033605 27657168

[53] Mantovani A., Allavena P., Sica A. & Balkwill F. Cancer-related inflammation. Nature 454, 436–444 (2008). 10.1038/nature07205 18650914

[54] Grivennikov S. I., Greten F. R. & Karin M. Immunity, inflammation, and cancer. Cell 140, 883–899 (2010). 10.1016/j.cell.2010.01.025 20303878 PMC2866629

[55] Liu T., Zhang L., Joo D. & Sun S. C. NF-kappaB signaling in inflammation. Signal Transduct Target Ther 2, 17023- (2017). 10.1038/sigtrans.2017.23 29158945 PMC5661633

[56] Qian Y., Kang Z., Liu C. & Li X. IL-17 signaling in host defense and inflammatory diseases. Cell Mol Immunol 7, 328–333 (2010). 10.1038/cmi.2010.27 20514051 PMC4002676

[57] Medzhitov R. Origin and physiological roles of inflammation. Nature 454, 428–435 (2008). 10.1038/nature07201 18650913

[58] Harris N. L. E. SerpinB2 regulates stromal remodelling and local invasion in pancreatic cancer. Oncogene 36, 4288–4298 (2017). 10.1038/onc.2017.63 28346421 PMC5537606

[59] Shuman Moss L. A., Jensen-Taubman S. & Stetler-Stevenson W. G. Matrix metalloproteinases: changing roles in tumor progression and metastasis. Am J Pathol 181, 1895–1899 (2012). 10.1016/j.ajpath.2012.08.044 23063657 PMC3506216

[60] Yang Y. The Apelin/APLNR system modulates tumor immune response by reshaping the tumor microenvironment. Gene 834, 146564 (2022). 10.1016/j.gene.2022.146564 35598689

[61] Ehira N. An embryo-specific expressing TGF-beta family protein, growth-differentiation factor 3 (GDF3), augments progression of B16 melanoma. J Exp Clin Cancer Res 29, 135 (2010). 10.1186/1756-9966-29-135 20950440 PMC2972255

[62] Pal B. A single-cell RNA expression atlas of normal, preneoplastic and tumorigenic states in the human breast. EMBO J 40, e107333 (2021). 10.15252/embj.2020107333 33950524 PMC8167363

[63] Mantovani A. The chemokine system in diverse forms of macrophage activation and polarization. Trends Immunol 25, 677–686 (2004). 10.1016/j.it.2004.09.015 15530839

[64] Zhang S. C1q(+) tumor-associated macrophages contribute to immunosuppression through fatty acid metabolic reprogramming in malignant pleural effusion. J Immunother Cancer 11 (2023). 10.1136/jitc-2023-007441 PMC10445384 37604643

[65] Yang J. Integrated genomic and transcriptomic analysis reveals unique characteristics of hepatic metastases and pro-metastatic role of complement C1q in pancreatic ductal adenocarcinoma. Genome Biol 22, 4 (2021). 10.1186/s13059-020-02222-w 33397441 PMC7780398

[66] Yang Q. Single-Cell RNA Sequencing Reveals the Heterogeneity of Tumor-Associated Macrophage in Non-Small Cell Lung Cancer and Differences Between Sexes. Front Immunol 12, 756722 (2021). 10.3389/fimmu.2021.756722 34804043 PMC8602907

[67] Chen L. NLRP3 in tumor-associated macrophages predicts a poor prognosis and promotes tumor growth in head and neck squamous cell carcinoma. Cancer Immunol Immunother 72, 1647–1660 (2023). 10.1007/s00262-022-03357-4 36586012 PMC10991898

[68] Daley D. NLRP3 signaling drives macrophage-induced adaptive immune suppression in pancreatic carcinoma. J Exp Med 214, 1711–1724 (2017). 10.1084/jem.20161707 28442553 PMC5461004

[69] Li F. L. INHBA promotes tumor growth and induces resistance to PD-L1 blockade by suppressing IFN-gamma signaling. Acta Pharmacol Sin 46, 448–461 (2025). 10.1038/s41401-024-01381-x 39223366 PMC11747416

[70] Burdick M. D., Harris A., Reid C. J., Iwamura T. & Hollingsworth M. A. Oligosaccharides expressed on MUC1 produced by pancreatic and colon tumor cell lines. J Biol Chem 272, 24198–24202 (1997). 9305871 10.1074/jbc.272.39.24198

[71] Hofmann B. T. COSMC knockdown mediated aberrant O-glycosylation promotes oncogenic properties in pancreatic cancer. Mol Cancer 14, 109 (2015). 10.1186/s12943-015-0386-1 26021314 PMC4447007

[72] Madsen C. B. Glycan elongation beyond the mucin associated Tn antigen protects tumor cells from immune-mediated killing. PLoS One 8, e72413 (2013). 10.1371/journal.pone.0072413 24039759 PMC3765166

[73] Medzhitov R. The spectrum of inflammatory responses. Science 374, 1070–1075 (2021). 10.1126/science.abi5200 34822279

[74] Bruni D., Angell H. K. & Galon J. The immune contexture and Immunoscore in cancer prognosis and therapeutic efficacy. Nat Rev Cancer 20, 662–680 (2020). 10.1038/s41568-020-0285-7 32753728

[75] Thorsson V. The Immune Landscape of Cancer. Immunity 48, 812–830 e814 (2018). 10.1016/j.immuni.2018.03.023 29628290 PMC5982584

[76] van der Meijs N. L., Travecedo M. A., Marcelo F. & van Vliet S. J. The pleiotropic CLEC10A: implications for harnessing this receptor in the tumor microenvironment. Expert Opin Ther Targets 28, 601–612 (2024). 10.1080/14728222.2024.2374743 38946482

[77] Geijtenbeek T. B. & Gringhuis S. I. C-type lectin receptors in the control of T helper cell differentiation. Nat Rev Immunol 16, 433–448 (2016). 10.1038/nri.2016.55 27291962

[78] Biswas S. K. & Mantovani A. Macrophage plasticity and interaction with lymphocyte subsets: cancer as a paradigm. Nat Immunol 11, 889–896 (2010). 10.1038/ni.1937 20856220

[79] Mills C. D., Lenz L. L. & Harris R. A. A Breakthrough: Macrophage-Directed Cancer Immunotherapy. Cancer Res 76, 513–516 (2016). 10.1158/0008-5472.CAN-15-1737 26772756 PMC4738030

[80] Leone R. D. & Powell J. D. Metabolism of immune cells in cancer. Nat Rev Cancer 20, 516–531 (2020). 10.1038/s41568-020-0273-y 32632251 PMC8041116

[81] van der Meijs N. L. Ligand-specific tuning of CLEC10A signalling strength and dendritic cell responses through engagement of different GalNAc-containing glycan structures. FEBS J (2025). 10.1111/febs.70317 41241848

[82] Macauley M. S., Crocker P. R. & Paulson J. C. Siglec-mediated regulation of immune cell function in disease. Nat Rev Immunol 14, 653–666 (2014). 10.1038/nri3737 25234143 PMC4191907

[83] Andrews S. FastQC: A Quality Control Tool for High Throughput Sequence Data. Available online at: http://www.bioinformatics.babraham.ac.uk/projects/fastqc. (2010).

[84] Kim D., Paggi J. M., Park C., Bennett C. & Salzberg S. L. Graph-based genome alignment and genotyping with HISAT2 and HISAT-genotype. Nat Biotechnol 37, 907–915 (2019). 10.1038/s41587-019-0201-4 31375807 PMC7605509

[85] Liao Y., Smyth G. K. & Shi W. featureCounts: an efficient general purpose program for assigning sequence reads to genomic features. Bioinformatics 30, 923–930 (2014). 10.1093/bioinformatics/btt656 24227677

[86] Love M. I., Huber W. & Anders S. Moderated estimation of fold change and dispersion for RNA-seq data with DESeq2. Genome Biol 15, 550 (2014). 10.1186/s13059-014-0550-8 25516281 PMC4302049

[87] Xu S. Using clusterProfiler to characterize multiomics data. Nat Protoc 19, 3292–3320 (2024). 10.1038/s41596-024-01020-z 39019974

[88] Pal B. A single-cell RNA expression atlas of normal, preneoplastic and tumorigenic states in the human breast. Embo j 40, e107333 (2021). 10.15252/embj.2020107333 33950524 PMC8167363

[89] Hao Y. Dictionary learning for integrative, multimodal and scalable single-cell analysis. Nat Biotechnol 42, 293–304 (2024). 10.1038/s41587-023-01767-y 37231261 PMC10928517

[90] McGinnis C. S., Murrow L. M. & Gartner Z. J. DoubletFinder: Doublet Detection in Single-Cell RNA Sequencing Data Using Artificial Nearest Neighbors. Cell Syst 8, 329–337 e324 (2019). 10.1016/j.cels.2019.03.003 30954475 PMC6853612

[91] Nofech-Mozes I., Soave D., Awadalla P. & Abelson S. Pan-cancer classification of single cells in the tumour microenvironment. Nat Commun 14, 1615 (2023). 10.1038/s41467-023-37353-8 36959212 PMC10036554

[92] Hu C. CellMarker 2.0: an updated database of manually curated cell markers in human/mouse and web tools based on scRNA-seq data. Nucleic Acids Res 51, D870–D876 (2023). 10.1093/nar/gkac947 36300619 PMC9825416

[93] Cheng S. A pan-cancer single-cell transcriptional atlas of tumor infiltrating myeloid cells. Cell 184, 792–809 e723 (2021). 10.1016/j.cell.2021.01.010 33545035

[94] Chen Y., Chen L., Lun A. T. L., Baldoni P. L. & Smyth G. K. edgeR v4: powerful differential analysis of sequencing data with expanded functionality and improved support for small counts and larger datasets. Nucleic Acids Res 53 (2025). 10.1093/nar/gkaf018 PMC11754124 39844453

